# Comparative Analysis of Intestinal Microflora Between Two Developmental Stages of *Rimicaris kairei*, a Hydrothermal Shrimp From the Central Indian Ridge

**DOI:** 10.3389/fmicb.2021.802888

**Published:** 2022-02-15

**Authors:** Li Qi, Chun-Ang Lian, Fang-Chao Zhu, Mengke Shi, Li-Sheng He

**Affiliations:** ^1^Institute of Deep-Sea Science and Engineering, Chinese Academy of Sciences, Sanya, China; ^2^College of Earth Sciences, University of Chinese Academy of Sciences, Beijing, China; ^3^Key Laboratory of Tropical Marine Ecosystem and Bioresource, Fourth Institute of Oceanography, Ministry of Natural Resources, Beihai, China

**Keywords:** intestine microflora, hydrothermal vent, *Rimicaris kairei*, *Deferribacteraceae*, *Sulfurovum*

## Abstract

Despite extreme physical and chemical characteristics, deep-sea hydrothermal vents provide a place for fauna survival and reproduction. The symbiotic relationship of chemotrophic microorganisms has been investigated in the gill of *Rimicaris exoculata*, which are endemic to the hydrothermal vents of the Mid-Atlantic Ridge. However, only a few studies have examined intestinal symbiosis. Here, we studied the intestinal fauna in juvenile and adult *Rimicaris kairei*, another species in the *Rimicaris* genus that was originally discovered at the Kairei and Edmond hydrothermal vent fields in the Central Indian Ridge. The results showed that there were significant differences between juvenile and adult gut microbiota in terms of species richness, diversity, and evenness. The values of Chao1, observed species, and ASV rarefaction curves indicated almost four times the number of species in adults compared to juveniles. In juveniles, the most abundant phylum was Deferribacterota, at 80%, while in adults, Campilobacterota was the most abundant, at 49%. Beta diversity showed that the intestinal communities of juveniles and adults were clearly classified into two clusters based on the evaluations of Bray–Curtis and weighted UniFrac distance matrices. *Deferribacteraceae* and *Sulfurovum* were the main featured bacteria contributing to the difference. Moreover, functional prediction for all of the intestinal microbiota showed that the pathways related to ansamycin synthesis, branched-chain amino acid biosynthesis, lipid metabolism, and cell motility appeared highly abundant in juveniles. However, for adults, the most abundant pathways were those of sulfur transfer, carbohydrate, and biotin metabolism. Taken together, these results indicated large differences in intestinal microbial composition and potential functions between juvenile and adult vent shrimp (*R. kairei*), which may be related to their physiological needs at different stages of development.

## Introduction

Deep-sea hydrothermal vents are distributed along mid-ocean ridges and are characterized by high temperatures and environments that are sulfide- and iron-rich, with a low pH (White et al., 2006; Möller et al., 2017). Despite the hostile environment, a large number of microorganisms and macroorganisms can be found in the hydrothermal area. The shrimp *Rimicaris exoculata* is one of the dominant macroorganisms in the hydrothermal vents of the Mid-Atlantic Ridge (MAR) and can reach densities of up to 3,000 individuals per square meter in the mixing zone of hydrothermal fluid and the surrounding cold, oxygenated seawater (Gebruk et al., 2000).

In deep-sea hydrothermal vents, symbiosis between macroorganisms and chemoautotrophic microorganisms is a common phenomenon (Goffredi et al., 2004; Goffredi, 2010; Watsuji et al., 2015; Hinzke et al., 2019). *R. exoculata* has been reported to coexist with branchiostegite chemoautotrophic microorganisms (Zbinden et al., 2008; Petersen et al., 2010; Hügler et al., 2011; Guri et al., 2012; Jan et al., 2014). Microbial symbionts benefit from the relatively stable habitat at the interface between electron donors and receptors for energy metabolism and provide organic carbon in return to their hosts. In contrast, only a few studies have been focused on the intestinal microflora of macroorganisms in hydrothermal vents, although intestinal microflora play important roles in diverse physiological events, such as nutrient absorption and immune protection, in almost all vertebrates and invertebrates outside vents (Dick, 2019). Van Dover et al. (1988) found that metallic sulfide crystals filled the stomach and gut of hydrothermal shrimp in the MAR. At the time, it was thought that they ate the sulfur compounds around the chimneys for nutrition. The presence of microorganisms in the gut of *R. exoculata* from the MAR was first observed by transmission electron microscopy in 2003; subsequent 16S rRNA analysis identified them as mainly Epsilonproteobacteria, Entomoplasmatales, and Deferribacterota (Zbinden and Cambon-Bonavita, 2003). Later, Durand et al. (2010) found that long-term starvation changed the dominant gut microflora of *R. exoculata*, supporting the hypothesis that a symbiotic relationship existed between *R. exoculata* and its gut epibionts. Durand et al. (2015) further defined the main lineage of resident gut epibionts at five hydrothermal vent locations and analyzed the relationship between the gut microbial communities and the different geographical locations. Apremont et al. (2018) reported the distribution, morphology, and phylogeny of microbial communities associated with the gut and gill of *Rimicaris chacei* in the MAR. The results showed that ε- and γ-proteobacteria were mainly found in the cephalothorax and digestive tract, and that Deferribacterota and Mollicutes were mainly found in the digestive tract. The microbial proliferation was explored during embryonic development of *R. exoculata* and increased with aged eggs (Methou et al., 2019).

*Rimicaris kairei* was discovered at the Kairei and Edmond fields in 2002, near the Rodriguez Triple Junction, Central Indian Ridge, Indian Ocean, at depths of 2,415–3,320 m (Watabe and Hashimoto, 2002). In this study, we comparatively characterized the gut microbial community of two developmental stages of the vent shrimp *R. kairei*, which further shed light on features of the intestinal microbiome in hydrothermal zones and provided basic information for functional analysis of the intestinal flora.

## Materials and Methods

### Sample Collection and Identification

The shrimps were collected at two hydrothermal vent fields on the Central Indian Ridge, Edmond (69.59667°E, 23.87782°N; 3,281 m depth) and Kairei (70.04010°E, 25.32048°N; 2,421 m depth), during the cruises on February 2019 by the research ship “Tan Suo Yi Hao.” All shrimps were obtained using the suction sampler from a manned submersible (“Shen Hai Yong Shi”). Once onboard, specimens were immediately frozen at −80°C or stored in 75% ethanol at −20°C.

According to body length, these shrimps were divided into two groups, defined as juveniles (<4 cm in length), and adults (>6 cm in length) (Jiang et al., 2020). Ten individuals of juvenile and ten individuals of adult were randomly selected from the two hydrothermalvents. Species of these shrimp were identified based on cytochrome *c* oxidase subunit I (*COI*). We identified the species as *R. kairei* (Supplementary Figure 1), based on the similarity of the *COI* sequence and the phylogenetic tree constructed through MEGA-X (64-bit) (Kumar et al., 2018) and Neighbor-Joining using Tamura 3-parameter model, with 1,000 bootstrap replications.

### DNA Extraction and Sequencing

The leg muscle (for *COI* sequence) and whole gut samples were isolated under sterile conditions. The gut before the fourth body segment is the foregut, while the rest constitute the hindgut (Zbinden and Cambon-Bonavita, 2003; Durand et al., 2010). DNA was extracted from each sample with the Power Soil DNA isolation kit (Qiagen, Hilden, Germany) following the manufacturer’s instructions. The V3–V4 region of 16S rRNA was amplified from the extracted DNA using the bacterial universal primers 341F: 5′-CCTAYGGGRBGCASCAG-3′ and 806R: 5′-GGACTACHVGGGTWTCTAAT-3 (Gonnella et al., 2016). Amplifications were performed on a Gene Amps PCR System 9700 (PE Applied Biosystems, Foster City, CA, United States) with the following program: 98°C for 30 s; 98°C for 15 s, 58°C for 15 s, and 72°C for 15 s, for a total of 30 cycles, followed by 72°C for 1 min. DNA was amplified in a 50 μl reaction composed of 25 μl Phusion High-Fidelity PCR Master Mix with HF Buffer, 3 μl DMSO, 0.3 μM of each primer, 50 ng DNA template, and nuclease-free water. The PCR products were purified with an AxyPrep DNA Gel Extraction Kit (Axygen, Union City, CA, United States) and quantified by Qubit fluorometric quantitation (Life Technologies, Carlsbad, CA, United States). Paired-end sequencing was performed on a NovaSeq PE-250 (Illumina) platform of GuHe Bioinformatics Technology (Hangzhou, China).

### Taxonomic Classification, Diversity Analysis, and Statistical Analysis

The V3–V4 sequences of 16S rRNA from a total of 40 samples were deposited in an SRA (Sequence Read Archive) database under accession numbers SRR16293710–SRR16293749. The datasets of microbiota from the vent environment were downloaded from SRA compared with the intestinal microbiota of *R. kairei*. Relative information on the vent environmental samples is summarized in Supplementary Table 1.

Analysis of the V3–V4 sequence was conducted with QIIME2 v2021.8 along with the built-in plugins (Bolyen et al., 2019). First, adapters at both ends of the sequences were removed using the q2-cutadapt plugin after demultiplex. Then, DADA2 (v1.16) (Callahan et al., 2016) was used for sequence trimming, denoising, and dereplication. Chimera sequences were also filtered. Paired reads were merged with a minimum length of 12 bp overlap. The ASV table was filtered out by q2-filter-feature after removing the ASVs with frequencies less than 5. Taxonomic classification was processed using the q2-classify-sklearn algorithm, and the SILVA (V.138) database was used as a reference with a threshold of 0.8. Annotations were obtained after removing contamination using the q2-feature-table plugin and visualized by the q2-taxa-barplot plugin. The ASVs annotated as mitochondria, chloroplasts, or eukaryotes were also removed.

Alpha diversities (Faith_phylogenetic diversity, Pielou’s evenness, observed_species, Chao1, Shannon and Simpson indices) and beta diversities (Bray–Curtis, weighted UniFrac, and unweighted UniFrac) were estimated using q2-diversity after normalization to 16,714 sequences per sample according to the minimum number of sequences in the samples. Kruskal–Wallis rank-sum tests were used to detect significant differences in alpha diversity. Beta diversities were visualized using non-metric multidimensional scaling (NMDS) and principal coordinate analysis (PCoA) plots. ANOSIM (analysis of similarities) was used to analyze the similarities of the microbial community compositions of the two groups.

### Identification of Featured Microbes

To identify the discriminative microbes of juvenile and adult groups, two methods were used: Random Forest and STAMP (v2.1.3). Random Forest is a machine learning classification algorithm that creates predictions by parallel learning of multiple tree predictors built randomly and trained on different subsets of data (Breiman, 2001). The ASV table with abundance was divided into 80:20 for training and testing. The ‘randomForest’ package in R 4.0.3 was used to classify the training data, specify juvenile and adult groups as classification variables, and calculate the importance of each ASV feature by assigning ‘importance = TRUE’. The out-of-bag (oob) error estimate of the error rate was 4%. Next, the test dataset and the key characteristic bacteria were predicted based on the value of mean decrease accuracy. According to the ‘confusionMatrix’ and ‘multiclass.roc’ functions, the accuracy of the confusion matrix was 93.33%, the balanced accuracy was 95%, and the area under the curve (AUC) value was 0.98. Finally, the 20 microbes with the highest mean decrease accuracy were selected and visualized by bar plots and heatmaps, using the ‘ggplot2’ package. Two-sided Welch’s tests with 95% confidence intervals were used in STAMP v2.1.3 and corrected by Benjamini–Hochberg FDR multiple tests (Parks et al., 2014; Li et al., 2021). The shared microbes resulting from the aforementioned approaches were selected and considered as featured microbes that discriminated juvenile and adult groups.

### Analysis of Potential Functions

The Phylogenetic Investigation of Communities by Reconstruction of Unobserved States (PICRUSt) 2.0 pipeline was used to predict the potential functions of the bacterial communities based on 16S rRNA sequences (Douglas et al., 2020). Sequences used in the PICRUSt analysis were first clustered into ASVs, with a similarity threshold of 0.99 in QIIME2 v2021.8 and using the Greengenes database (version 13.5) as a reference for clustering. The Kyoto Encyclopedia of Genes and Genomes (KEGG) annotation was performed by the KEGG Automatic Annotation Server (KAAS) with a bidirectional best-hit method and the representative genome set of prokaryotes. According to the KEGG database, KO level 3 was displayed within the KEGG pathway hierarchy. Kruskal–Wallis rank-sum test was used to evaluate the significance. Pearson correlations were used to estimate the relationship between metabolic pathways and featured bacteria. The plot was constructed using ggplots2 in R (v.4.0.3).

## Results

### Compositions of the Microbial Communities

The rarefactions for these studied samples were saturated and are displayed in Supplementary Figure 2. We obtained 1,280,230 raw sequences from the 40 samples, ranging from 16,714 to 40,375 per sample. After cleaning, a total of 1,265,852 high-quality reads were generated, with an average of 31,646 reads per sample. A total of 1,412 unique ASVs were identified and included in all downstream analyses. These ASVs were classified into 29 phyla and 340 genera.

**FIGURE 1:**
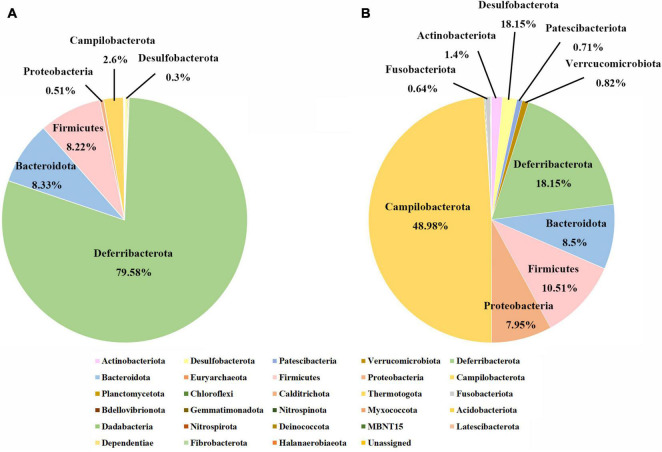
Gut microbial compositions of juvenile and adult samples at the phylum level. **(A)** The phylum level in the juvenile group; **(B)** the phylum level in the adult group.

The top 5 phyla in the juvenile group included Deferribacterota, Bacteroidetes, Firmicutes, Proteobacteria, and Campilobacterota, accounting for almost 100% of the total ASVs, and the predominant phylum was Deferribacterota, accounting for approximately 80% (Figure 1A). In the adult group, the most dominant phylum was Campilobacterota, with approximately 50% of the total ASVs. Deferribacterota, which accounted for approximately 80% of the total ASVs in juveniles, accounted for the second-largest proportion in adults, at 20%. The proportions of Bacteroidetes and Firmicutes in the juvenile and adult groups were similar, close to 10%. However, proteobacteria varied greatly between the adult and juvenile groups, occupying approximately about 8% in the adult group and only 0.5% in the juvenile group (Figure 1B). A total of 340 genera were identified, but there were only 44 genera with an abundance of more than 1% (Supplementary Figure 3). In juveniles, the largest group was *Deferribacteraceae*, accounting for 80% ± 16% (mean ± SD), followed by *Flavobacteriaceae*, which accounted for 8% ± 7%. In adults, the top genus was *Sulfurovum*, accounting for 40% ± 20%. The second one was *Deferribacteraceae*, which accounted for 17% ± 17%. Among these 44 genera, 34 showed significant differences in abundance between the whole gut bacteria of the two developmental groups, according to Wilcoxon rank-sum tests (Supplementary Table 2). Among these different genera, only *Deferribacteraceae* and *Tyzzerella* were more enriched in juveniles, while the other 32 genera were more enriched in adults. The genera with the greatest difference were *Deferribacteraceae* (juvenile vs. adult: 80% vs. 17%), and *Sulfurovum* (juvenile vs. adult: 2% vs. 40%) (Supplementary Figure 4).

### Structural Characteristics of the Intestinal Microbial Communities

**FIGURE 2:**
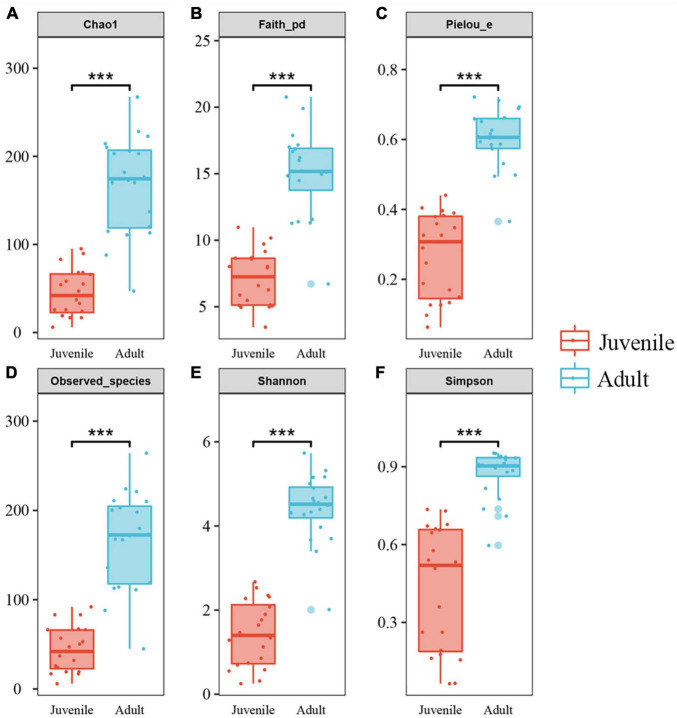
Alpha diversities of gut microbiota in the two developmental stages of *R. kairei*. **(A–F)**: Chao1, Faith_phylogenetic diversity, Pielou’s evenness, observed_species, Shannon and Simpson indices are displayed in the box plots (Non-parametric Kruskal–Wallis tests, ***p < 0.001). The middle lines in the boxes indicate the median. The upper and lower lines of the box indicate the upper and lower quartiles, respectively. The scattered dots represent the values of each sample. The larger dots represent outliers.

**FIGURE 3:**
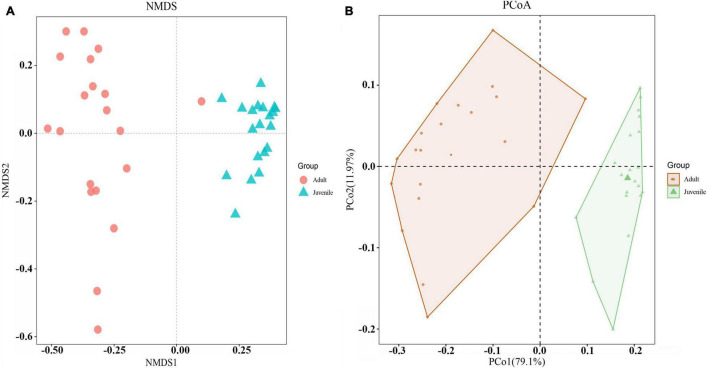
Beta diversities of the two gut microbial communities. **(A)** NMDS was calculated by Bray–Curtis distance for juvenile and adult shrimp (stress = 0.07). Red dots indicate adult samples. Green triangles indicate juvenile samples. **(B)** PCoA indicated the similarity of microbiota composition and the phylogenetic distance between the two groups based on the weighted UniFrac algorithm. Component axes indicate the degree of variance.

Species richness, diversity, and evenness were compared between the juvenile and adult groups. The results showed that the indices examined in this study were significantly higher in adults than in juveniles, indicating that the adult gut microbial community had more species, more diversity, and more evenness (Figure 2). For Chao1 and Observed species index, the adult group was more than four times higher than the juvenile group, which was consistent with the ASVs by rarefaction curves (Supplementary Figure 2). And for other indices, the adult group appeared at least two times of juveniles (Figure 2). The beta diversity analysis clearly revealed two clusters of juvenile and adult gut microbial communities (Figure 3). The adult gut microbial community was dispersed in the NMDS (non-metric multidimensional scaling) analysis (Figure 3A). Significant differences between the two bacterial communities were identified by the analysis of similarities (ANOSIM) based on Bray–Curtis distance (ANOSIM statistic R: 0.972, *p* = 0.001). In the PCoA, principal coordinate 1(PCo1) explained 79.1% of the variance (Figure 3B), R was 0.938 and *p* = 0.001 in ANOSIM analysis, indicating a significant difference between the juvenile and adult groups. In addition, the gut microbial community exhibited low richness, diversity, and evenness compared to the environmental samples (Supplementary Figures 5A–F). According to the beta diversity analysis, the gut community was clearly distinct from environmental samples but had a relatively high dispersion (ANOSIM: R = 0.485, *p* = 0.001) (Supplementary Figure 5G).

### Featured Gut Microbes in Juveniles and Adults

**FIGURE 4:**
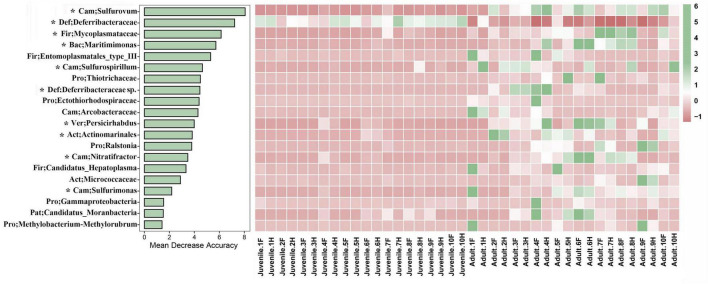
Random forest analysis was performed between the two different developmental stages of *R. kairei*. The bar plot shows the influence of the top 20 bacteria based on the accuracy of the random forest model. The heatmap shows the bacteria with normalized abundance in the two different groups. Cam, Campilobacterota; Def, Deferribacterota; Fir, Firmicutes; Bac, Bacteroidetes; Pro, Proteobacteria; Ver, Verrucomicrobia; Act, Actinobacteria; Pat, Patescibacteria. F, foregut; H, hindgut. * indicates the shared bacteria with STAMP.

To identify the featured microbes that differed between the juvenile and adult gut microbial communities, a random forest model was constructed. The top 20 genera with the highest variable importance are shown in Figure 4. Among these genera, only one (classified as *Deferribacteraceae*) was more abundant in juveniles than in adults; the other 19 genera were lower in juveniles. The top five most featured microbes included *Sulfurovum*, *Deferribacteraceae*, *Mycoplasmataceae*, *Maritinminonas*, and *Entomoplasmatales*_type_III. *Sulfurovum*, the top-ranking microbe, had a mean decrease accuracy (MDA) value of approximately 8 and showed high abundance in all adult samples. *Deferribacteraceae*, the second-ranking microbe, had an MDA value of 7 and the highest abundance in all juvenile samples, but was almost absent in most of the adult samples. The MDA values of *Mycoplasmataceae*, *Maritinminonas*, and *Entomoplasmatales*_type_III ranged from 6 to 5. In the STAMP analysis, 13 genera were filtered out (Supplementary Figure 6), 10 of which were shared with random forest analysis.

### Potential Functions of Gut Microbiota

**FIGURE 5:**
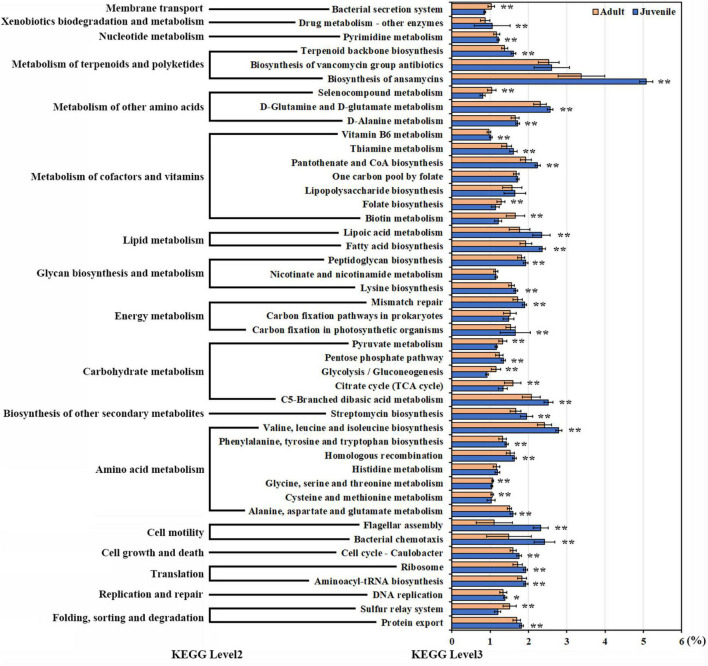
The pathways with significant differences between juveniles and adults. Pathways with more than 1% abundance were included in this differential analysis. The diverse pathways are displayed on the *y*-axis. Relative abundances are displayed on the *x*-axis. Orange bars indicate adult samples. Blue bars indicate juvenile samples. Asterisks indicate a significant difference. (Non-parametric Kruskal–Wallis tests, *p < 0.05, **p < 0.01).

Based on 16S rRNA, 45 pathways, each with more than 1% abundance, were predicted by PICRUSt2; of these, 39 pathways were significantly different between the juvenile and adult groups. These pathways were primarily concerned with metabolism, cell processes, genetic information processing, and environmental information processing. Most of the pathways displayed a higher abundance in juveniles, except pathways for glycolysis; the citrate cycle; the sulfur relay system; metabolism of glycine, serine, threonine, pyruvate, biotin, cysteine, methionine, and selenocompound; folate biosynthesis; and the bacterial secretion system, which were more abundant in adults. The biosynthesis of ansamycins and the cell motility pathway were enriched in the juvenile group. In addition, the pathways related to lipid metabolism and essential amino acid synthesis were also higher in juveniles (Figure 5). Correlation analysis was performed between 10 featured microbes and the pathways with significant differences between juveniles and adults. The results revealed that *Deferribacteraceae*, the featured bacteria in juveniles, was negatively correlated with the other nine featured microbes in adults in most of the different pathways (Supplementary Figure 7).

## Discussion

### Community Structures of Gut Microbiota Differed Between Juveniles and Adults

The assemblage of a resident microbial community is important for animal development. By investigating the gut microbial communities of juvenile and adult hydrothermal vent shrimp, we found that developmental stage is an important factor affecting the intestinal flora of *R. kairei*. This study showed that the adult gut microbial community had higher index values of species richness, diversity, and evenness than juveniles. This phenomenon has also been reported in other animals. In Malaysian mahseer (*Tor tambroides*), the gut microbiota showed higher diversity in the larval, juvenile, and adult stages than that in fingerling and yearling stages by a culturable approach (Mohd Nosi et al., 2018). The community structure of *Triatoma rubrofasciata* gut microbiota also appeared to be affected by aging, with increased species richness and evenness in aged individuals (Hu et al., 2020). This phenomenon applies not only to marine animals and insects but also to humans. Researchers have reported that healthy adults have significantly higher diversity of their gut microbiomes than young children in the United States (Ringel-Kulka et al., 2013). Similarly, a detailed comparison of the intestinal microbiota of 1-year-old infants and 4-year-old children in China revealed that the species richness, evenness, and diversity of the intestinal microbiota were significantly lower in the 1-year-olds (Guo et al., 2020). These aging effects not only occur in gut microbiota but also in other parts of the symbiotic microbiota. A study reported that the surface flora structure of *R. exoculata* embryos changed during development. The species diversity and evenness of the bacterial assemblages on egg and pleopod surfaces were higher in the late stage than in the early and middle stages (Methou et al., 2019). There are several possible explanations as to why aged gut flora are richer and more diverse. First, with aging, intestinal cells not only increase in number but also become more increasingly complex in morphology and physiological functions. The diverse intestinal cells and diverse intestinal microenvironment affect the growth and composition of attached microorganisms (Bergh, 1995). Stephens et al. (2016) reported that the morphological changes of intestinal cells during development may be the main driver of changes in the intestinal microbiome, investigating 135 zebrafish intestinal microbial communities from developmental periods given the same diet and environmental conditions (Stephens et al., 2016). Second, adult individuals tend to have more complex diets and living conditions than juveniles, resulting in changes in intestinal microbial diversity. Jin et al. (2021) evaluated the effects of dietary changes, habitat changes, and lifestyle shifts on the gut microbiota of giant pandas with high-throughput sequencing and genome-resolved metagenomics. The results showed that high-fiber diets significantly increased the species diversity and decreased the richness of gut bacterial communities (Jin et al., 2021). Third, the habitat can also greatly affect bacterial communities. Although it is currently unclear whether the habitats of *R. kairei* adults and juveniles are the same, it has been suggested that hydrothermal shrimp may migrate from a few 100 m above the hydrothermal vents to the area around the chimneys, according to the isotopic trace of carbon found in *R. exoculata* (Methou et al., 2020). External environmental conditions can certainly affect diet. In the alpha diversity results of this study, Chao 1 indicated that species richness exhibited the greatest difference between juveniles and adults, with an almost fourfold change. Species diversity and evenness differed between these developmental stages. The changes in intestinal microflora community indicated that their role changes between developmental stages of *R. kairei*.

### Potential Functions of Gut Microbiota in Juvenile and Adult Shrimp

According to the PICRUST results, many metabolic pathways exhibited differences between adult and juvenile *R. kairei*. Although ansamycin biosynthesis was the most abundant metabolic pathway in both juvenile and adult groups, the relative abundance of ansamycin synthesis pathways in juvenile compared to other pathways was almost 1.5-fold that in adults. Carbon metabolic and sulfur transfer pathways were substantially more abundant in the adult group. These results indicate that the gut microbiota of juvenile individuals includes more antibacterial functions to protect their hosts from the invasion of pathogenic bacteria, while the gut microbiota of adults is more related to energy metabolism.

The most featured bacterial group in the guts of juveniles was *Deferribacteraceae*, which was also a main lineage found in the gut of other vent shrimp (Durand et al., 2010; Apremont et al., 2018). The functions of the phylum Deferribacterota have received less attention, perhaps because most of them are uncultivable. The most studied genus is *Deferribacter*, which inhabits deep and shallow seas and includes four species: *Deferribacter thermophilus*, *Deferribacter desulfuricans*, *Deferribacter abyssi*, and *Deferribacter autotrophicus*. Of these, *D. desulfuricans* and *D. autotrophicus* are the only two species with an available complete genome and whose possible metabolic pathways have been reported. The novel anaerobic heterotrophic bacterium *D. desulfuricans* SSM1 was isolated from a deep-sea hydrothermal vent chimney at the Suiyo Seamount of the Izu-Bonin Arc, Japan (Takai et al., 2003). Genomic annotation and comparison revealed that many of its genes were similar to sulfur-reducing or sulfate-reducing bacteria in the phylum Deltaproteobacteria. Analysis of metabolic pathways revealed that the bacterium was capable of using a variety of organic acids, such as formate, acetate, and pyruvate, as carbon sources. This genome also encodes chemoreceptors, chemotaxis-like systems, and signal transduction machinery, suggesting that the bacterium possesses versatile energy metabolism for surviving its extreme environments (Takaki et al., 2010). In contrast, *D. autotrophicus* is a thermophilic chemolithoautotrophic anaerobe that is capable of CO_2_ fixation by the roTCA cycle and that couples the oxidation process of CO with nitrate reduction using anaerobic [Ni, Fe]-containing CO dehydrogenase, the first carbon monoxide oxidation process identified in the phylum Deferribacterota (Slobodkin et al., 2019). A Nap-type complex encoding nitrate reduction was also identified, which may be involved in Fe(III) reduction. Deferribacteraceae, along with Muribaculaceae and Lachnospiraceae, was also the dominant family present in mouse intestines and was associated with cofactor, vitamin, and amino acid metabolism (Chung et al., 2020). Another study reported that the relative abundance of *Deferribacter* spp. and *Spirochaetes* spp. in the gut microbial community of horses increased after 160 h of dietary treatment with moxidectin (Daniels et al., 2020). Taken together, it is hypothesized that *Deferribacteraceae* in the juvenile gut may be important for host health and adaptation to extreme environments.

The genus *Sulfurovum* belongs to the family *Sulfurovaceae*. Most strains of this genus grow chemolithoautotrophically using sulfur as an energy source. Representatives of this genus include *Sulfurovum lithotrophicum* (Inagaki et al., 2004), *Sulfurovum riftiae* (Giovannelli et al., 2016), *Sulfurovum* sp. (Nakagawa et al., 2007), *Sulfurovum aggregans* (Mino et al., 2014), *Sulfurovum denitrificans* (Mori et al., 2018), and *Sulfurovum indicum* (Xie et al., 2019). Most of them are from lithological samples, except *S. riftiae*, which was isolated from the hydrothermal tubeworms. Among them, only *S. lithotrophicum* and *S. denitrificans* can use oxygen as the electron acceptor; the others can use nitrate, sulfur, or thiosulfate as the electron acceptor. All of these species mainly use hydrogen, sulfur, or thiosulfate as the electron donors. As an environmental bacterium, *Sulfurovum* sp. NBC37-1 utilized hydrogen-oxidizing sulfur respiration and thiosulfate-oxidizing nitrate/oxygen respiration for sulfur-related energy metabolism (Nakagawa et al., 2007; Lösekann et al., 2008). *Sulfurovum* sp. NBC37-1 can use elemental sulfur as both electron acceptor and donor, which allows this class of microorganisms to adapt to both highly reduced hydrothermal and oxygen-rich environments (Yamamoto et al., 2010). *S. riftiae* is an anaerobic, nitrate-reducing, sulfur- and thiosulfate-oxidizing bacterium, which uses CO_2_ as the only carbon source and nitrate as the only terminal electron acceptor (Giovannelli et al., 2016). A recent metagenomic study on the hydrothermal vent shrimp *R. exoculata* revealed that the reductive tricarboxylic acid (rTCA) and Calvin–Benson–Bassham (CBB) cycles were used for carbon fixation by two filamentous epibionts belonging to Campylobacteria and Gammaproteobacteria, respectively. These epibionts could couple the oxidation of reduced sulfur compounds or molecular hydrogen to oxygen or nitrate reduction (Jan et al., 2014). *Sulfurovum* was the most abundant genus in the adult guts of *R. kairei*. At present, the understanding of the intestinal flora of hydrothermal shrimp is unclear. *Sulfurovum* lineage exists in both gill and intestinal tract besides environments; some researchers speculated that the bacteria in the intestinal tract may belong to the transient community retained by permanent feeding (Zbinden and Cambon-Bonavita, 2020). A recent study on the symbiosis of gills in five different hosts in a hydrothermal environment found that *Sulfurovum* is an opportunistic combination with weak host selectivity (Lee et al., 2021). In the guts of *R. kairei*, there is more than 100 ASVs in the genus *Sulfurovum* and the top 10 ASVs in abundance accounted for 88.7%. None of the 10 ASVs was assigned as a known species with cutoff of 0.8. Guts provide an anaerobic or hypoxic environment. Strains in *Sulfurovum* are facultative anaerobes or anaerobes. Moreover, metallic sulfide crystals filled the stomach and gut of hydrothermal shrimp (Van Dover et al., 1988; Zbinden and Cambon-Bonavita, 2003). All of these suggest that it is possible for *Sulfurovum* to grow in guts, even as symbionts, and to be involved in sulfur cycle and other functions.

### Possible Horizontal Transmission of Gut Microbiota in *R. kairei*

Like all other arthropods, *R. exoculata* undergoes molts, which regularly eliminate the bacterial community that has settled on the cuticle (Corbari et al., 2008). In contrast, the gut has no cuticle layer, and therefore, the gut surface does not renew during molting. The symbionts of this area were therefore supposedly maintained throughout the lifecycle of the animal following their acquisition (Durand et al., 2010). Regarding life history, isotopic data have been used to argue that *R. exoculata* has a long planktotrophic larval dispersal stage before it settles on hydrothermal vents and transitions to the chemosynthetic feeding pattern of juveniles (Gebruk et al., 2000). Lipids and stable carbon isotope analyses of *R. exoculata* indicated that these animals possess a high level of polyunsaturated fatty acids, which can be mobilized to enable growth and maturation of the vent shrimp at a suitable site (Pond et al., 2000). Previous studies have shown that *R. exoculata* harbors two symbioses: epibiotic communities located at branchiostegites, including a wide diversity of Epsilon-, Gamma-, Alpha-, Beta-, Delta-, Zetaproteobacteria, and Bacteroidetes (Zbinden et al., 2008; Petersen et al., 2010; Hügler et al., 2011; Guri et al., 2012; Jan et al., 2014; Cambon-Bonavita et al., 2021), and bacterial communities colonized in guts, which are composed of Deferribacteres, Mollicutes, Campylobacteria, and, to a lesser extent, Gammaproteobacteria (Zbinden and Cambon-Bonavita, 2003; Durand et al., 2010, 2015). These results are consistent with the intestinal microflora of *R. kairei* from the CIR in this study, which is also mainly composed of Deferribacteres, Mollicutes, and Campylobacteria. However, Bacteroidetes was the major phylum in the guts of *R. kairei* from the CIR, but that was not reported in the guts of *R. exoculata* from MAR. Tyzzerella, belonging to the Lachnospiraceae family, in a relatively high proportion in the juvenile group, was not reported in the guts of *R. exoculata* from MAR. All of these suggest that though *R. kairei* and *R. exoculata* belong to hydrothermal shrimp, the microorganism community in their guts appeared different. And the different composition may be affected by geographical environment, species, and others. Although the same species, gut microbial community still showed difference to some extent, indicating the importance of the environmental factor. Cowart et al. (2017) analyzed the communities in three MAR hydrothermal vents across two organs (digestive tract and stomach), three molting colors (white, red, and black), and three life stages (egg, juvenile, and adult) using cluster network analysis. The results showed that the OTUs of Epsilonproteobacteria were geographically segregated, and proposed a combination of transmission modes including environmental selection and vertical inheritance for the symbiont of hydrothermal shrimp *R. exoculata* (Cowart et al., 2017). In the current study, of the 44 genera with abundances above 1%, 36 genera showed significant differences in abundance across the whole gut microbiota between the two groups. These 36 significantly different genera occupied approximately 90 and 80% of the total composition in juveniles and adults, respectively. Taken together with the diversity and discriminative flora analysis, this indicates that there may be horizontal transmission in the gut microbiota of *R. kairei*. Whether there is vertical propagation is uncertain and would require more information related to embryos.

## Conclusion

There were significant differences in the gut microflora structure, such as species richness, diversity, and evenness, between juvenile and adult *R. kairei*. These results suggest that the presence of gut microbial variation across two developmental stages could be an adaptation strategy for both symbionts and hosts in extreme environments.

## Data Availability Statement

The datasets presented in this study can be found in online repositories. The names of the repository/repositories and accession number(s) can be found in the article/Supplementary Material.

## Author Contributions

LQ and L-SH conceived and designed the experiments and wrote the manuscript with input from all other authors. LQ, C-AL, F-CZ, and MS performed the experiments and analyzed the data. L-SH directed and supervised all of the research. All authors contributed to the article and approved the submitted version.

## Conflict of Interest

The authors declare that the research was conducted in the absence of any commercial or financial relationships that could be construed as a potential conflict of interest.

## Publisher’s Note

All claims expressed in this article are solely those of the authors and do not necessarily represent those of their affiliated organizations, or those of the publisher, the editors and the reviewers. Any product that may be evaluated in this article, or claim that may be made by its manufacturer, is not guaranteed or endorsed by the publisher.
