# Supplementary material for: Comparative Analysis of Intestinal Microflora Between Two Developmental Stages of Rimicaris kairei, a Hydrothermal Shrimp From the Central Indian Ridge
Source: Front Microbiol. 2022 Feb 15;12:802888. doi: 10.3389/fmicb.2021.802888 (PMC8886129; doi:10.3389/fmicb.2021.802888)
Supplement: Supplementary file 1 [file Data_Sheet_1.docx]

**Table S1 Information of environment samples**

| Sample ID | Sampling sites | Longitude | Latitude | Depth (m) | BioProject accession number | Type |
| --- | --- | --- | --- | --- | --- | --- |
| S1 | SWIR | 54.36841 | 34.802 | 3272.35 | SRR8632127 | sediment; deep-sea surface sediment |
| S2 | SWIR | 54.2143 | 34.96707 | 3269.63 | SRR8632128 | sediment; deep-sea surface sediment |
| S3 | SWIR | 54.2267 | 34.87032 | 3492.67 | SRR8632125 | sediment; deep-sea surface sediment |
| S4 | SWIR | 54.6153 | 34.84631 | 2335.7 | SRR8632126 | sediment; deep-sea surface sediment |
| TVG4 | SWIR | 50.9277 | 37.6251 | 2086 | SRR2231135 | sediment; deep-sea surface sediment |
| TVG11 | SWIR | 50.9643 | 37.6174 | 1985 | SRR2231136 | sediment; inactive hydrothermal vents |
| SAP-1_S | South Atlantic | 14.4 | 13.2 | 2334 | SRR10061075 | seawater; Hydrothermal rising plume |
| SAP-2_S | South Atlantic | 14.4 | 13.2 | 2312 | SRR10061074 | seawater; Hydrothermal neutrally buoyant plume |
| SAP-3_S | South Atlantic | 14.4 | 13.2 | 2293 | SRR10061070 | seawater; Hydrothermal neutrally buoyant plume |
| SAP-4_S | South Atlantic | 14.4 | 13.2 | 2261 | SRR10061069 | seawater; Hydrothermal neutrally buoyant plume |
| SAP-5_S | South Atlantic | 14.4 | 13.2 | 2273 | SRR10061068 | seawater; Hydrothermal neutrally buoyant plume |
| S4_S | South Atlantic | 14.6 | 13.6 | 3751 | SRR10061064 | sediment; Hydrothermal sediments |
| S7_S | Southwest Indian Ocean | 49 | 37.4 | 3282 | SRR10061063 | sediment; Hydrothermal sediments |
| S8_S | Southwest Indian Ocean | 52.2 | 37.5 | 3982 | SRR10061073 | sediment; Hydrothermal sediments |
| S21_S | South Atlantic | 13.3 | 15.2 | 3271 | SRR10061072 | sediment; Hydrothermal sediments |
| SMAR | South Atlantic | 14.5 | 13.8 | 2933 | SRR10061067 | sediment; Hydrothermal sulfide (active sites) |
| SWIP | Southwest Indian | 49.6 | 37.8 | 3126 | SRR10061065 | sediment; Hydrothermal sulfide (active sites) |

**Table S2 Genera with significant difference between juveniles and adults**

| **Genus** | **Juvenile**  **Average abundance (%)** | **Adult**  **Average abundance (%)** | ***p* value** | **FDR** |
| --- | --- | --- | --- | --- |
| Cam;Sulfurovum | 2.250073838 | 40.21123119 | ** | 6.38E-09 |
| Def;Deferribacteraceae | 80.49863128 | 17.31034005 | ** | 6.06E-09 |
| Fir;Mycoplasmataceae | 0.810173604 | 8.610575838 | ** | 3.71E-05 |
| Cam;Arcobacteraceae | 0.079971331 | 5.107454308 | ** | 6.24E-05 |
| minorgroup | 0.270118673 | 3.883111913 | ** | 4.47E-09 |
| Des;Desulfobulbus | 0.135820014 | 1.10008673 | ** | 0.000954 |
| Bac;Maritimimonas | 0.053648791 | 1.090840632 | ** | 0.000323 |
| Cam;Sulfurospirillum | 0.084670388 | 1.088377009 | ** | 6.36E-05 |
| Cam;Sulfurimonas | 0.029999649 | 1.044758722 | ** | 3.73E-05 |
| Pro;Cardiobacteriaceae | 0.050724483 | 1.043367162 | ** | 0.000672 |
| Fir;Entomoplasmatales_type_III | 0.03652588 | 0.775294723 | ** | 0.001717 |
| Fus;Psychrilyobacter | 0.092525202 | 0.633045992 | ** | 0.002671 |
| Ver;Persicirhabdus | 0.046579934 | 0.579196772 | ** | 0.000333 |
| Des;Desulfocapsa | 0.149313259 | 0.535536952 | ** | 0.011869 |
| Pro;Cocleimonas | 0.072157216 | 0.529727302 | ** | 0.000283 |
| Pro;Mariprofundus | 0.12693435 | 0.495889694 | * | 0.028492 |
| Pat;Candidatus_Moranbacteria | 0.116549063 | 0.488468611 | * | 0.041233 |
| Pro;Undibacterium | 0.011993088 | 0.466739567 | ** | 0.010255 |
| Pro; PS1_clade | 0.001356807 | 0.43199479 | ** | 0.005771 |
| Fir;Candidatus_Hepatoplasma | 0.02343586 | 0.431828762 | ** | 0.000297 |
| Cam;Nitratifractor | 0.023256796 | 0.330735477 | ** | 0.001879 |
| Pro;Cupriavidus | 0.002968691 | 0.316560041 | ** | 0.012691 |
| Def;Deferribacteraceae sp. | 0.003876863 | 0.303486131 | ** | 0.001683 |
| Cam;Campylobacter | 0.019940629 | 0.272935543 | ** | 0.003106 |
| Fir;Streptococcus | 0.007340799 | 0.266083063 | * | 0.028492 |
| Act;Actinomarinales | 0.013986654 | 0.252190913 | ** | 0.000237 |
| Fir;Lysinibacillus | 0.015205778 | 0.223041764 | * | 0.026439 |
| Pro;Sphingomonadaceae | 0.000313322 | 0.212952059 | * | 0.042687 |
| Bac;Carboxylicivirga | 0.049924688 | 0.207208491 | * | 0.055612 |
| Pro;Thiotrichaceae | 0.002005218 | 0.178868594 | ** | 0.000211 |
| Bac;Elizabethkingia | 0.00166992 | 0.135505118 | ** | 0.002387 |
| Pro;Caulobacteraceae | 0.001912692 | 0.098710761 | ** | 0.006786 |
| Pro;Methylobacterium-Methylorubrum | 0.000379987 | 0.093191255 | ** | 0.002551 |
| Fir;Tyzzerella | 4.449284735 | 0.091510924 | * | 0.060436 |
| Pro;Ectothiorhodospiraceae | 0.000864723 | 0.083608906 | ** | 0.002671 |

**p*<0.05; ***p*<0.01
