# Supplementary material for: Comparative Analysis of Intestinal Microflora Between Two Developmental Stages of Rimicaris kairei, a Hydrothermal Shrimp From the Central Indian Ridge
Source: Front Microbiol. 2022 Feb 15;12:802888. doi: 10.3389/fmicb.2021.802888 (PMC8886129; doi:10.3389/fmicb.2021.802888)
Supplement: Supplementary file 2 [file Data_Sheet_2.docx]

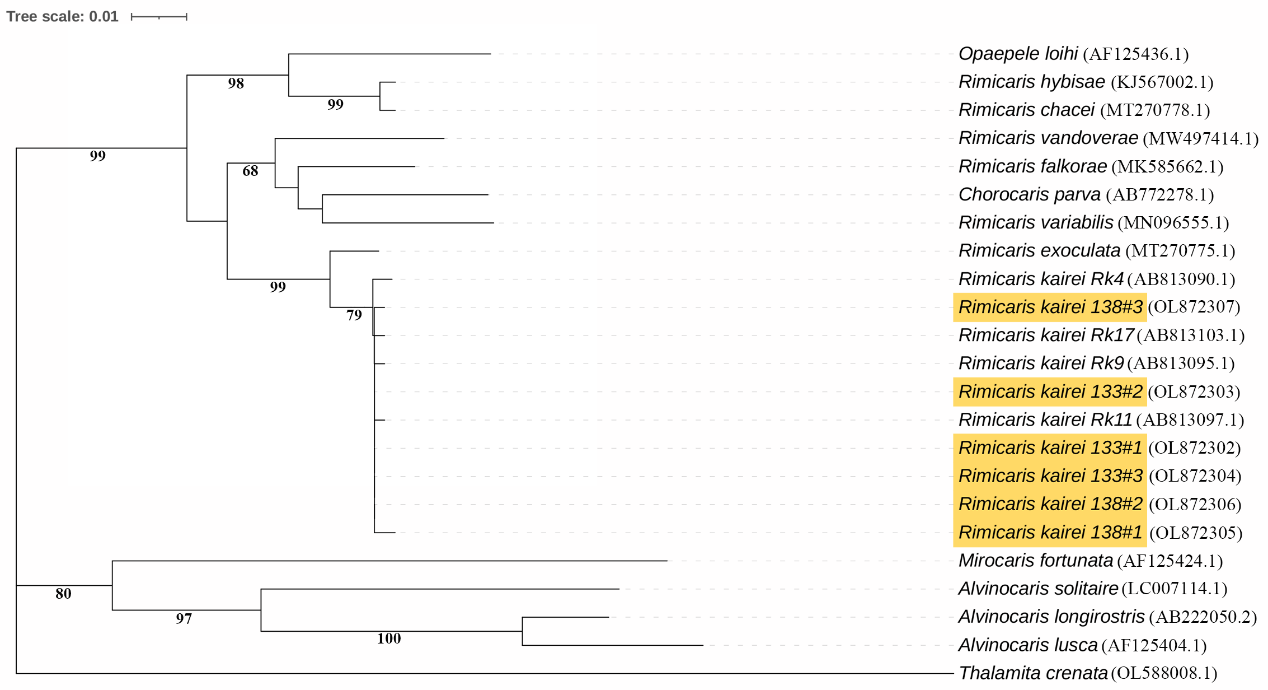


**Fig S1.** Phylogenetic tree based on *COI* sequences by Neighbor-Joining approach. *Thalamita crenata* crab are used as outgroup. The sequences highlighted in orange are from this study. Bootstrap values are indicated on the branches, and accession numbers are also labeled.


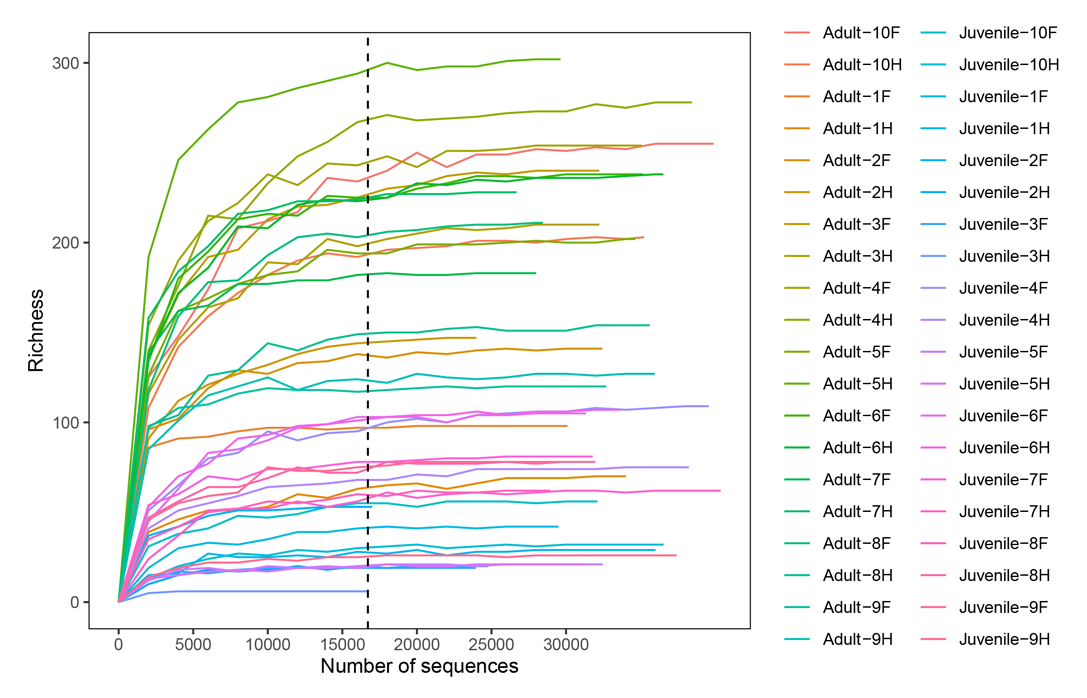


**Fig. S2** Rarefaction curves of juvenile and adult gut microbial samples. F: foregut; H: hindgut.


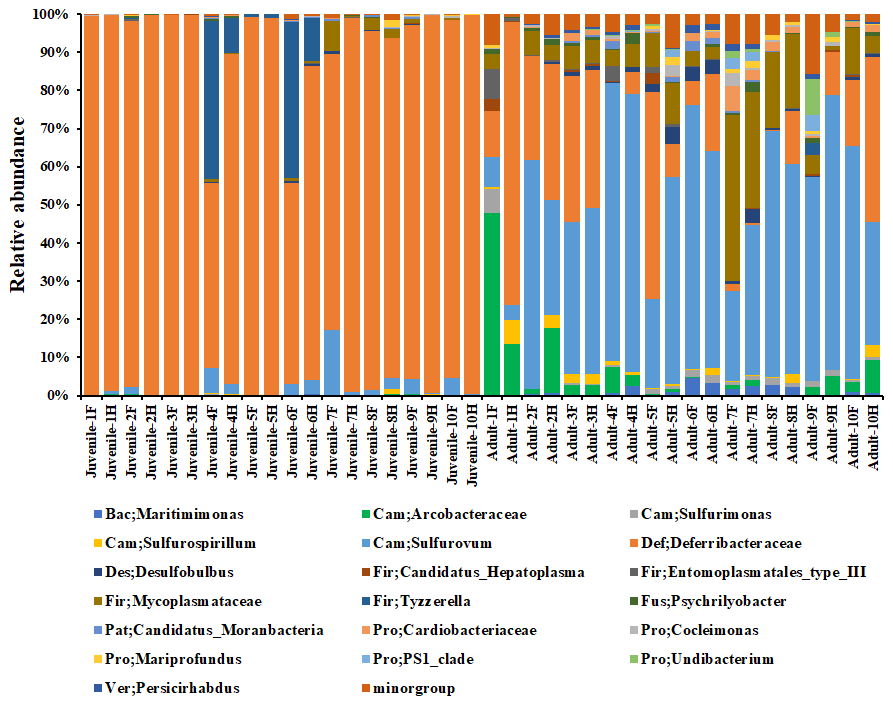


**Fig. S3** Gut microbial compositions of juvenile and adult *R.kairei* at the top20 genus level. The microbes with abundance less than 1% were classified in a minor group. F, foregut; H, hindgut. Bac, Bacteroidetes; Cam, Campilobacterota; Def, deferribacterota; Fir, Firmicutes; Fus, Fusobacteria; Pat, Patescibacteria; Pro, Proteobacteria; Ver, Verrucomicrobia.


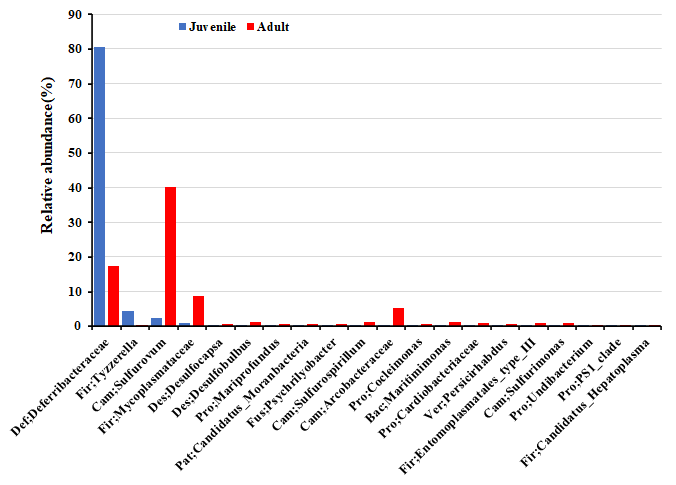


**Fig. S4** Top 20 genera ranked by their contributions to the difference between the Juvenile and Adult groups (Wilcox test, *p* < 0.05). The horizontal axis represented the different genera. The vertical axis represented the relative abundance. Blue bar: Adults; Red bar: Juveniles.

**
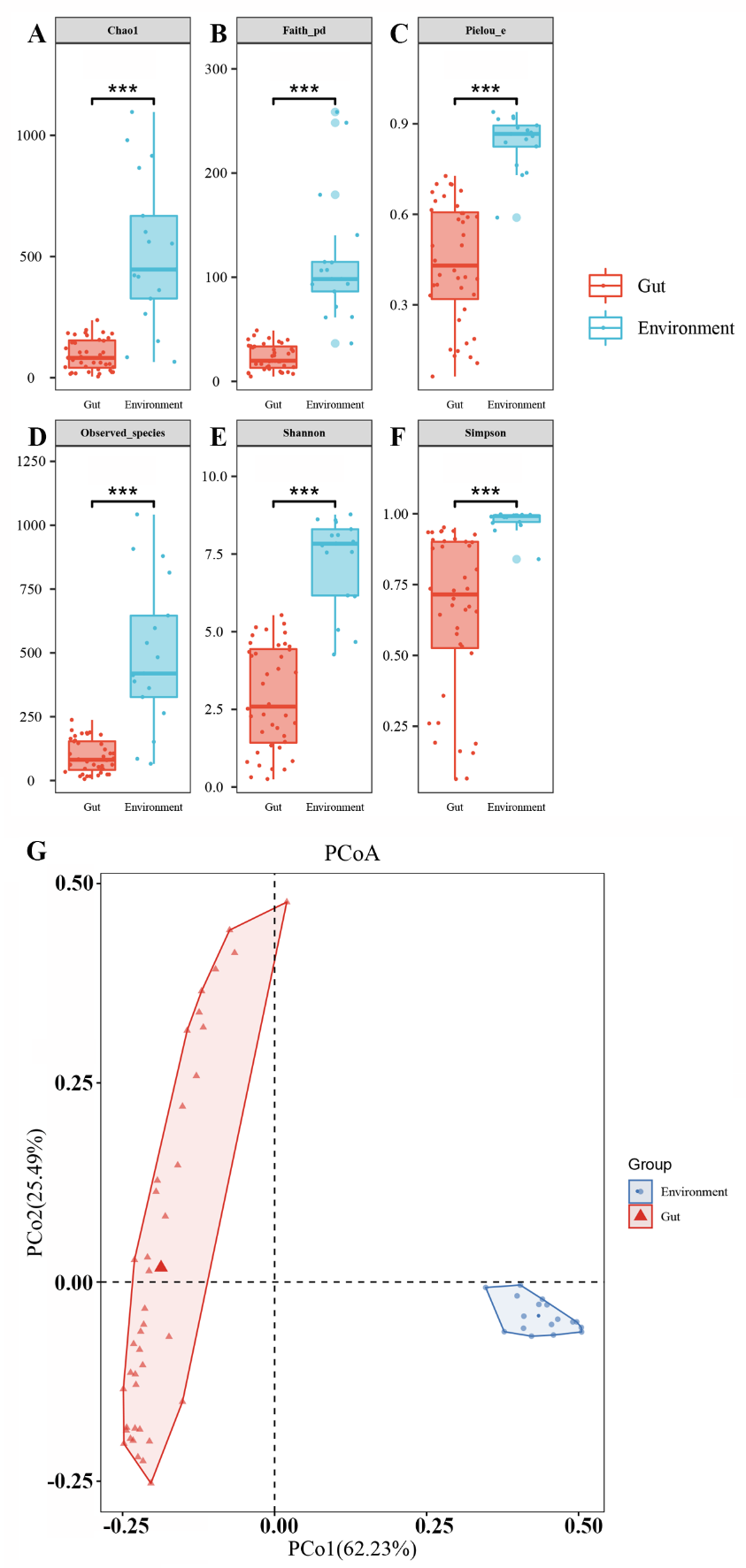
**

**Fig. S5** Alpha and beta diversities in gut and environment samples. (A)-(F): Chao1, Faith_phylogentic diversity, Pielou’s evenness, Observed_species, Shannon, Simpson and were displayed by box plot, respectively. (Nonparametric Kruskal- Wallis test, *** p<0.001). (G) PCoA analysis based on Unweighted UniFrac algorithm indicated the similarity of microbiota composition and closeness of phylogenetic distance in all samples (ANOSIM: *R* = 0.982, *p* = 0.001). Component axes indicate explaining degree of variance.


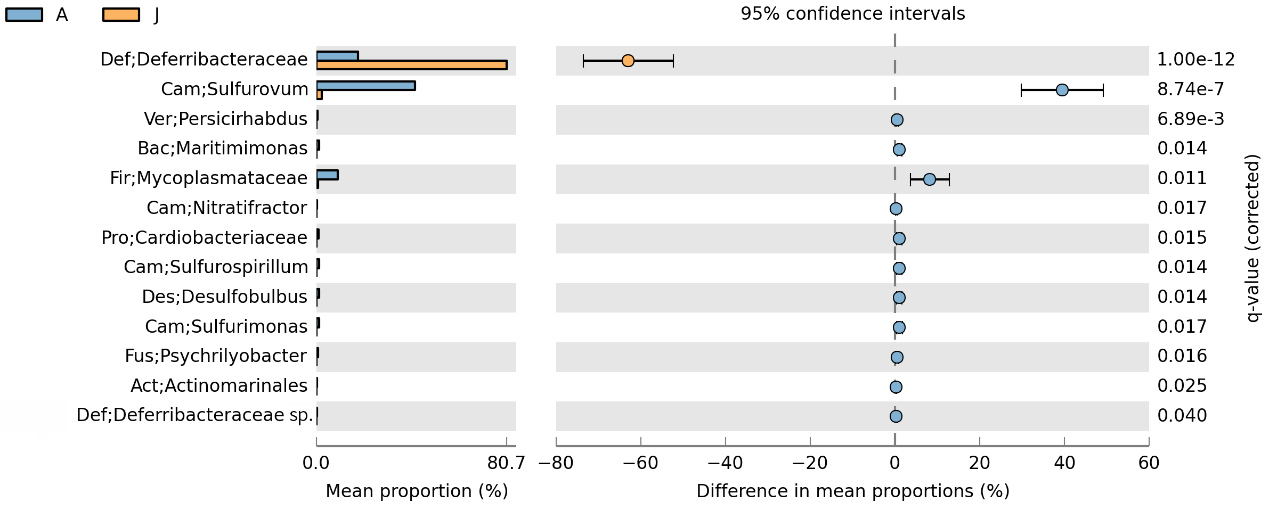


**Fig. S6** STAMP analysis of juvenile and adult groups at genus level (Welch’t-test/confidence interval, 0.95/*p*<0.05). Blue bar: adults; Orange bar: Juveniles. A: adult; J: juvenile.


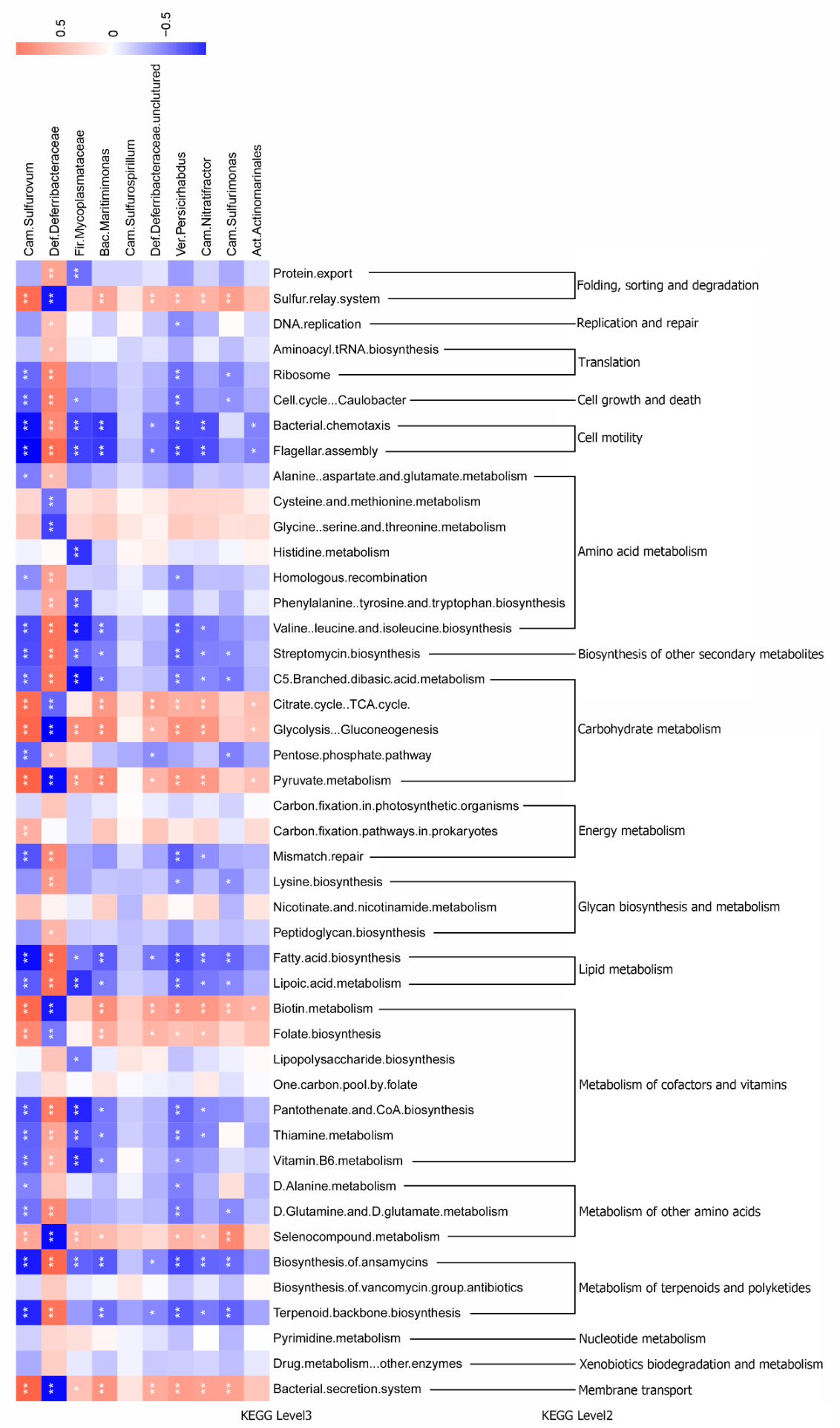


**Fig. S7** Correlations between the featured microbes and the pathways. Color depth indicated the degree of Pearson correlation coefficients, red indicated the positive correlation, and blue indicates the negative correlation (**p<*0.05; ***p<*0.01).
